# Psychometric properties of the external Housing-Related Control Belief Questionnaire among people with Parkinson’s disease

**DOI:** 10.1007/s40520-020-01477-4

**Published:** 2020-02-07

**Authors:** Nilla Andersson, Maria H. Nilsson, Björn Slaug, Frank Oswald, Susanne Iwarsson

**Affiliations:** 1 Department of Health Sciences, Lund University, Lund, Sweden; 2 Clinical Memory Research Unit, Department of Clinical Sciences Malmö, Lund University, Lund, Sweden; 3 Memory Clinic, Skåne University Hospital, Malmö, Sweden; 4 Interdisciplinary Ageing Research, Goethe University, Frankfurt, Germany

**Keywords:** Psychometrics, Control beliefs, Parkinson’s disease, Reliability, Validity

## Abstract

**Background:**

Housing-related control beliefs are associated with aspects of health among older people in general. Research on Parkinson’s disease (PD) focusing on perceptions of the home are rare and instruments capturing perceived aspects of home have seldom been used.

**Aims:**

To evaluate psychometric properties of the external Housing-related Control Beliefs Questionnaire (HCQ) among people with PD.

**Methods:**

The external HCQ were administrated to 245 participants with PD, (mean age = 69.9 years; mean PD duration = 9.7 years). External HCQ has 16-items, with five response options. The psychometric properties evaluated were data quality, structural validity (factor analysis), floor and ceiling effects, corrected item total correlations, internal consistency and construct validity (testing correlations with relevant constructs according to pre-defined hypotheses).

**Results:**

Data quality was high. Structural validity showed a unidimensional construct with removal of two items. Homogeneity was questionable, but strengthened after the removal of the two items. For the 14-item version internal consistency was α = 0.78 and SEM 4.47. Corrected item total correlation ranged between 0.31 and 0.54 and no floor or ceiling effects. Significant correlations with relevant constructs supported the construct validity.

**Conclusions:**

Taken together, the psychometric results suggest a 14-item version of the external HCQ to be sufficiently reliable and valid for use in the PD population. The results pave the way for further studies, using the HCQ to analyse how perceptions of control of the home may be associated with health among people ageing with PD.

## Introduction

A basic methodological prerequisite is that the data collection instruments are evaluated psychometrically in the targeted populations. In this study, we evaluated the external Housing-related control beliefs Questionnaire (external HCQ) [1-4] for use among people with Parkinson’s disease (PD).

The transaction between a person and his/her immediate home environment is dynamic, comprising of both objective environmental and perceived aspects of housing [5]. One perceived aspect of importance for maintenance of independence and autonomy [6] in old age is control beliefs. This is related to the concept locus of control [7] and has been regarded in relation to different outcomes such as cognitive functioning, daily independence and housing [1-4, 8]

Housing-related control beliefs address how people think about how they can deal with daily issues within their immediate home environment. Examining age-related differences and changes in housing-related control beliefs can improve the understanding of the mechanisms underlying the achievement and maintenance of autonomy and well-being in old age in general [4] and in particular with respect to changing autonomy [1, 3]. Oswald and colleagues developed an instrument to capture the concept that is the external HCQ Questionnaire [4]. The external HCQ instrument was introduced and psychometrically evaluated in two studies with community-dwelling older adults (sample 1, *N* = 485, 66–69 years; sample 2, *N* = 107; 65–91 years) [4]. In this evaluation, (internal consistency and test–retest reliability) the external HCQ instrument had two sub-scales, “powerful others” (sample 1: *α* = 0.66; sample 2: *α* = 0.72, *r*_tt_ = 0.78), and “chance” (sample 1: *α* = 0.83; sample 2: *α* = 0.76, *r*_tt_ = 0.50). The two sub-scales were also found to correlate with higher age, lower education, and lower income [4]. The 16 items of the two sub-scales were later combined, with results suggesting sufficient internal consistency (*α* = 0.72) [1]. Later studies have indicated associations between the external HCQ and ADL, life satisfactions, depression and psychological well-being [1, 9-11].

PD is a chronic neurodegenerative disease, with symptoms that worsen over time and with increasing complexity [12]. It often results in ADL difficulties, even at an early stage of the disease [13]. For example, up to 75% have gait and balance problems, complex activities such as home maintenance and shopping are more difficult to perform for people with PD than for compared controls [13]. There seems to be a shift from internal to external locus of control [7] for this group, as a strategy to manage a sense of lost control with the unpredictable and progressive disease [14]. When it comes to housing conditions, people with PD more often move to assisted living facilities [12, 15] and at an earlier age compared to the population in general [15]. This has a great impact for people living with PD including their families, and implies high societal costs [12]. To the best of our knowledge only two studies have used the external HCQ instrument in PD samples [16, 17]. Comparing housing aspects between people with self-reported PD (*N* = 20) and matched controls (*N* = 60), Nilsson et al. found no significant difference concerning external HCQ [16]. Further, they explored associations between aspects of health and perceived housing and found that people with PD rely on external control in relation to housing [17], which are in line with studies among community-dwelling older people [1]. Since, psychometric properties are sample dependent [18] and the external HCQ instrument has not been evaluated among people with PD it is uncertain how valid these results are.

Therefore, the aim of this study was to examine the psychometric properties of the external HCQ instrument to identify whether it validly and reliably capture external housing-related control beliefs among people with PD. We examined data quality, structural validity, floor and ceiling effects, corrected item total correlations, internal consistency reliability, convergent validity and known group validity.

## Methods

### Participants and recruitment

We used baseline data collected for the longitudinal cohort study “Home and Health in People Ageing with Parkinson’s Disease” (HHPD) in 2013. The design and procedures of the HHPD were described in detail in a study protocol [19]. Participants were recruited from three hospitals in Skåne County (i.e., study district). The inclusion criterion was being diagnosed with PD (G20.9) since at least one year. The exclusion criteria were difficulties understanding or speaking Swedish, having cognitive difficulties (evaluated by a specialized PD-nurse and screening of medical records) or other reasons making the individual unable to give informed consent (i.e., hallucinations or a recent stroke) or to take part in the majority of the data collection. Out of 653 possible participants, 58 lived outside the study district and were not included. Further, 158 were excluded having difficulties understanding or speaking Swedish (*n* = 10); severe cognitive problems (*n* = 91) or other reasons that made them unable to give informed consent or take part in the majority of the data collection (e.g. hallucinations, recent stroke) (*n* = 57). The remaining 437 potential participants were invited to participate. However, 22 of those were unreachable and two had their diagnosis changed. Furthermore, 157 out of 413 remaining participants declined and one had extensive missing data (*n* = 255). For this study, another eight persons were excluded due to comprehensive missing data on core variables and two more were excluded as they lived in residential care units. Thus, the final study sample was *N* = 245 (60.8% men; mean age = 69.9 years; min–max = 45–93 years) and the median duration with PD was 8 years (q1-q3 = 5–13) (Table 1). Additional descriptive data was collected, which is presented and described as footnotes in Table 1, capturing, i.e. cognitive [20], motor functioning [21] and housing variables.

**Table 1:** Sample characteristics, *N* = 245

Variable, *n* (%) unless otherwise stated	Descriptive	Missing, *n*
Participant characteristics		
Sex		
Women/men	96 (39.2)/149 (60.8)	0
Age, mean (SD)	69.7 (9.0)	1
Parkinson’s duration years, median (q1-q3)	8 (5–13)	1
Disease severity (HY^a^ in on)		
HY I	50 (20.4)	0
HY II	73 (29.8)	0
HY III	62 (25.3)	0
HY IV	54 (22)	0
HY V	6 (2.5)	0
Motor symptoms (UPDRS^b^ III), median (q1–q3)	29 (22–39)	4
Cognitive function (MOCA)^c^, median (q1–q3)	26 (23–28)	5
ADL^d^ (ADL Staircase), median (q1–q3)	4 (0–8)	0
Higher education (university), yes/no	83 (33.9)/ 162 (66.1)	0
Life satisfaction (Lisat -11, 1 item), median (q1–q3)	5, (4–5)	0
Housing characteristics		
Type of housing		
Apartment	109 (44.5)	0
Housing	131 (53.5)	0
Other	5 (2)	0
Residential location		
Rural	79 (32.2)	0
Semi-Urban	65 (26.5)	0
Urban	101 (41.3)	0
Tenure of housing		
Privately owned/rental	184 (75.1)/61 (24.9)	0
Accessibility problems (HE)^e^, median (q1–q3)	185 (95–272)	1
Housing adaptation, yes/no	80 (32.7)/165 (67.3)	0
Years in present dwelling, median (q1–q3)	17 (5–35)	0

^a^Hoehn and Yahr, eligible scores 1–5

^b^Unified Parkinson’s disease rating scale, eligible scores = 0–108

^c^Montreal cognitive assessment, 0–30

^d^Activities of daily living, eligible scores 0–27. 9 items that are rated from 0–3 (0 = independent–3 = dependent) and summed to a total sum score. Higher scores= the more dependent

^e^Housing Enabler, eligible scores 0–1844

Written informed consent was obtained from each participant and the study was conducted in accordance with the Helsinki Declaration. The Regional Ethical Review Board in Lund (No. 2012/558) approved the HHPD.

### Data collection

Two project administrators underwent project-specific training and completed the data collection. Data was collected through phone interview, postal survey and a subsequent home visits. The external HCQ instrument was administrated at the home visit.

### Measures

#### External Housing-related control beliefs (HCQ) instrument

The external HCQ instrument captures external control beliefs in relation to the home, such as help from others or chance/luck affecting the perceived control [1-4]. It is interview-administered and comprises of 16 items, each with five response options (1 = strongly disagree, 5 = strongly agree) [3]. The sum score ranges from 16 to 80 points (higher scores = indicate lower perceived control) [1, 2]. A multi-lingual research team engaged in an iterative translation process, which resulted in the Swedish version of the instrument used in the present study.

#### Construct validity hypotheses and variables used

Based on previous research [1, 22] and clinical reasoning, convergent and known group validity were assessed using predefined hypotheses (see Table 3, left column), including four variables identified as relevant: Disease severity was assessed according to the five-stage Hoehn and Yahr (HY) scale [23] in the “on-state” (range I–V, higher = worse).

Housing accessibility was assessed with the Housing Enabler (HE) instrument [24], administrated in three steps. The first step consists of dichotomous assessments of functional limitations and dependence on mobility devices and the second step is dichotomous assessments of physical environmental barriers. In a third step combining data from previous steps, a matrix-generated score is computed. A higher score indicates greater accessibility problems (max score = 1,844) [24].

ADL dependence was evaluated through interviews using the ADL Staircase [25, 26], which consists of nine items divided into personal ADL (P-ADL; feeding, transfer, toileting, dressing and bathing) and instrumental ADL (I-ADL; cooking, transportation, shopping and cleaning). Each assessment was rated on a three-graded scale: independent/partly dependent/dependent. If rated as independent, the participants was asked to state if the activity was done with or without difficulties [27]. A total sum score of the ADL Staircase was used (range 0–27), the higher the scores the more dependent.

The self-administrated General Self-Efficacy Scale [28] instrument consists of 10 items; each with four response options (ranging from “not at all true” to “exactly true”). The total sum score range from 10 to 40 points; the higher scores—the greater self-efficacy. The scale has been psychometrically evaluated among people with PD, with satisfactory results [29].

### Data analysis and statistical analysis

Participant characteristics were described using median, quartiles and frequencies, and psychometric properties were evaluated as described below.

Data quality was calculated for each item/total scale, i.e. the percentage of missing data for items and total scores [18], without imputation. Structural validity was investigated with exploratory factor analysis. Because the data was ordinal and not normally distributed, we used a principal axis factoring. Visual examinations of the scree plot and the factor loadings were used to evaluate the number of factors to include in the model [30]. Floor and ceiling effects were calculated for the total score [31] using the recommended 15–20% upper limit for interpretation of the results [18]. The distribution of response alternatives was examined to detect floor and ceiling effects on item level. While seemingly high, for interpretation we used 75% limit, which to the best of our knowledge is the only published recommendation available [32]. Internal consistency reliability was evaluated with Cronbach’s alpha, using values > 0.70 as acceptable [18]. The standard error of measurement (SEM) was calculated and complemented with a 95% confidence interval [18]. Corrected item-total correlation was investigated, considering values > 0.3 to indicate the correctness of summing the items to a total score [18]. To examine whether the items evaluate the same underlying construct, correlations > 0.4 were considered [18]. Convergent and known group validity were analysed with Spearman’s rang correlation and Mann–Whitney* U* test, respectively [33]. The level of statistical significance was set to *p* < 0.05.

The IBM SPSS statistics 25 software (IBM Corporation, Armonk, NY, USA) and the HE software (Veten & Skapen HB and Slaug Enabling Development, Lund and Staffanstorp, Sweden) were used for data and statistical analysis.

## Results

### Data quality

The median (q1-q3) total scores of the 16 item external HCQ instrument was = 38 (32–47). The overall data quality was high, with low proportions of missing item responses: 0.4% for two items and 0.8% for one (Table 2).

**Table 2:** Results of psychometric analyses for the 16- and 14-item versions of the external HCQ instrument, *N* = 245

External HCQ ^a^ instrument item	Median (q1–q3)	Missing (*n*)	Floor effect, *n* (%)	Ceiling effect, *n* (%)	Corrected item—total correlation	
			Response options		16 items	14 items
			“Strongly disagree”	“Strongly agree”		
I rely on others for helpful improvement in my home	3 (1–4)	0	68 (27.8)	16 (6.5)	0.35	0.34
Having a nice place is luck. You cannot influence it	2 (1–3)	0	109 (44.5)	14 (5.7)	0.45	0.45
Whether I will stay in my home, depend on other people	3 (2–4)	0	46 (18.8)	20 (8.2)	0.39	0.36
It’s luck if my neighbours will step in, if I need help	2 (1–3)	0	87 (35.5)	13 (5.3)	0.39	0.38
To do anything interesting outside my home, I rely on others	2 (1–4)	0	109 (44.5)	30 (12.2)	0.48	0.50
Whether or not I can stay in my home depends on luck and circumstance ^c^	4 (4–5)	0	21 (8.6)	114 (46.5)	0.24	–
I rely on others, to use the support services and community facilities	2 (1–4)	0	106 (43.3)	31 (12.7)	0.36	0.38
You have to live with the way your home is. You can’t do anything about it	2 (1–4)	0	100 (40.8)	30 (12.2)	0.38	0.39
When people offer to help, I can’t say no	2 (1–3)	2	107 (44.0)	23 (9.5)	0.48	0.49
Where and how I live, has happened by chance ^c^	3 (1–5)	0	97 (39.6)	70 (28.6)	0.27	–
Others have told me how to arrange the furnisher in my home	1 (1–2)	0	160 (65.3)	17 (6.9)	0.36	0.36
It is luck if I can continue my way of life, in my home in the future	3 (2–4)	1	56 (23.0)	37 (15.2)	0.56	0.53
I listen to advice from others, not to change anything in my home	2 (1–4)	1	94 (38.5)	20 (8.2)	0.33	0.32
The way my home has been set up, has happened by chance	4 (2–5)	0	42 (17.1)	65 (26.5)	0.33	0.34
Other people are to blame if my home is not a place where I can enjoy life	1 (1–2)	0	180 (73.5)	8 (3.3)	0.44	0.45
If there are support services or community facilities, depends on luck	2 (1–4)	0	98 (40.0)	24 (9.8)	0.32	0.31
External HCQ, 16-item version						
Total sum score, median (q1–q3)	38 (32–47)	4	1 (0.4)	0 (0.0)	Cronbach alpha/SEM^b^	
Total mean score, mean (SD)	2.52 (0.66)	4			0.78/4.97	
External HCQ, 14-item version						
Total sum score, median (q1–q3)	32 (26–39)	4	2 (0.8)	0 (0.0)	Cronbach alpha/SEM	
Total mean score, mean (SD)	2.28 (0.69)	4			0.78/4.47	

^a^Housing-related Control Belief Questionnaire, score range 1–5

^b^Standard error of measurement

^c^Item removed for the 14-item version

### Structural validity

The scree plot indicated that there were few factors. Using the criteria mentioned in the method section (testing one, two and three factor solutions), the results showed that the one-factor solutions was the best fit, explaining 19.8% of the variance. Due to the uni-dimensionality of the construct, rotation was not possible. The items “Whether or not I can stay in my home depends on luck and circumstance” and “Where and how I live has happened by chance”, had low corrected item correlations (< 0.3) and low factor loadings (< 0.32) [34]. Analysis excluding these items resulted in a one-factor solution with similar results for the remaining 14 items, explaining 21.4% of the variance.

### Floor and ceiling effects

Concerning the total score, no floor (0.4%) or ceiling effects were found (0%). On item level, there was tendency to floor effect for some. For details, see Table 2.

### Corrected item total correlations

The corrected item total correlations varied between 0.24–0.56. Five items had values over 0.4 and 2 items had values < 0.3. Two items (Table 2) with values < 0.3 were removed, resulting in improved corrected item total correlations. In the 14-item version, all 14 items were > 0.3 and five > 0.4.

### Internal consistency reliability

Cronbach’s alpha was 0.78 and SEM 4.97 (95% CI, -4.73 to 14.67). For the 14-item version, Cronbach’s alpha remained unchanged and SEM was 4.47 (CI 95%, −4.29 to 13.23) (Table 2).

### Convergent and known group validity

Regarding the known group validity, the group comparisons showed a significant difference between HY stages I-III and HY IV-V (Table 3). The convergent validity analysis showed that lower external HCQ correlated significantly with living in a dwelling with a lower magnitude of accessibility problems. Higher external HCQ correlated significantly with being dependent in ADL and lower general self-efficacy. Running the analysis for the 14-item version yielded roughly the same results.

**Table 3:** Hypotheses and results of the correlations between external HCQ instrument and differences in Parkinson’s disease severity, accessibility problems, dependence in ADL and general self-efficacy, *N* = 245

Hypotheses	Statistical result	Hypothesis confirmed
External HCQ^a^ expected to be significantly lower for HY^b^ I–III, than HY IV–V	Median HCQ: HY I–III = 37.0, HY IV–V = 47.5, *p* < 0.001	Yes
Higher external HCQ expected to significantly correlate with living with more accessibility problems^c^ (*r*_s>_0.37^d^)	*r*_s_ 0.48, *p* < 0.001	Yes
Higher external HCQ expected to significantly correlate with being dependent in ADL^e^ (*r*_s_ > 0.26)	*r*_s_ 0.41, *p* < 0.001	Yes
Higher external HCQ expected to significantly correlate with lower general self-efficacy^f^	*r*_s_ -0.35, *p* < 0.01	Yes

^a^Housing-Related Control Belief Questionnaire

^b^Hoehn and Yahr

^c^Housing Enabler

^d^Spearman’s correlation coefficient

^e^Activities of daily living

^f^General Self-Efficacy Scale

## Discussion

This study showed that the external HCQ instrument generates data of high quality and has acceptable internal consistency reliability with no floor and ceiling effects among people with PD. The homogeneity is questionable due to somewhat low corrected item total values, but improved after the removal of two items. The 14-item external HCQ instrument seems to represent a unidimensional construct, and the corrected item total correlations indicate that the items can be summed to a total score [18]. Correlations with relevant variables are in line with predefined hypotheses (Table 3) and support the construct validity of the external HCQ instrument.

To the best of our knowledge, analysis of homogeneity or factor analysis have not previously been done regarding the external HCQ instrument. Our results show that two items (Table 2) are not sufficiently correlated to the other items in the instrument, which motivated their removal. This resulted in corrected item total values above the recommended value [18] for all items. However, the percentages of the variance explained by the factor models are low, indicating that the instrument does not capture the complex concept of the external housing-related control beliefs in its entirety. It could also indicate that some questions are not optimally formulated, which is a consideration further supported by the tendency to limited response distribution for some items. That is, some expressions used may not be optimal to capture true variation. Yet, the analysis of structural validity is an important step forward to increase the understanding of the concept of housing-related control beliefs. It is challenging to capture such a complex concept in its entirety when using data collection instruments.

As to internal consistency reliability, Cronbach’s alpha (α = 0.78) is acceptable in this study [18] and slightly higher than in general populations of older people [1, 2]. This value remained unchanged in the 14-item version, supporting the use of the external HCQ instrument in the PD population. The measurement error of the external HCQ instrument has not previously been reported, and this new knowledge is important for longitudinal studies on home and health dynamics and studies of effects of housing interventions, etc. The SEM results are just below 5 points, which is a value that should be exceeded to indicate a real change [18]. It should be noted that this does not certify that a 5-point change is of clinical relevance. SEM can be influenced by different factors, such as the number of items, missing responses and vague instructions [35].

The results of this study are congruent with previous findings showing associations between the external HCQ instrument and with ADL, life satisfaction, depression and psychological well-being [1, 9-11]. As to convergent validity, our results are in agreement with the predefined hypothesis. The correlations with accessibility problems and dependence in ADL are slightly stronger than in previous studies [1]. Although theoretically, housing is only one of several specific domains of the concept of control beliefs [36]. The wider concept general control beliefs is a good predictor of quality of life, but not to predict how people cope with challenges in specific domains [37]. Research shows that with age the control over work, finances and marriage tends to increase, whereas control decreases in relation to relationships with children and sex life [36]. The fact that no recent studies have addressed these associations highlights the need for further research on these intriguing relationships.

In the PD population, research on housing has typically addressed interventions such as home modifications [38], while there a few studies aiming to increase the knowledge about how environmental aspects are associated with health. Few studies have focused on home and health dynamics in a comprehensive manner, and to deepen the knowledge about person-environment relationships the diversity of housing aspects warrants more research attention. To the best of our knowledge, the external HCQ instrument has only been used in two studies [16, 17]. Results from such studies give important information on housing aspects and associations to independence and autonomy [6], which is useful for rehabilitation and development of housing interventions and policies.

### Study limitations

The external HCQ instrument was developed as an unpublished German version and first assessed in the Interdisciplinary Longitudinal Study on Adult Development [39]. Later, it was translated to English [4] and Swedish [3]. We used the Swedish version but then used the English version for presentation in this paper. Overall, translations of instruments into different languages are challenging and might result in differences regarding psychometric properties. Although back translation often is considered the golden standard for instrument translation processes, its value or utility has been questioned. Back translation is not free from error and does not necessarily give a clear indication of the quality of a forward translation [40] Thus, Oswald et al. [3] engaged a multi lingual research team in an iterative translation process, which resulted in a Swedish version of the HCQ instrument of good quality. However, we cannot rule out that translation aspects underlie some of the differences in relation to previous studies evaluating the instrument.

Further, the percentages of the variance explained by the factor models are low, indicating that the instrument does not capture the complex concept of the external housing-related control beliefs in its entirety. This could also indicate that some questions are not optimally phrased, which is a consideration further supported by the tendency to limited response distribution for some items. In other words, some expressions used may not be optimal to capture true variation.

It is important to use psychometrically sound instruments in both research and practice [35]. However, a psychometric evaluation is an on-going progress and additional studies are warranted. A limitation in this study is that test–retest reliability was not possible to evaluate, because the HHPD project was not designed to evaluate test–retest reliability. Thus, the database did not include the data required for such evaluation. Accordingly, that kind of analysis was not feasible within the HHPD project. To compare test–retest values would have been interesting, as Oswald et al. showed high to medium results for the original external HCQ sub-scales [3].

## Conclusions

This study indicates that the reliability and construct validity of the external HCQ instrument is sufficient, and supports the use of the 14-item version in the PD population. The structural validity of the instrument is not optimal due to the low explanation of the variance of the complex concept of housing-related control beliefs. Although, external HCQ instrument seems to represent a unidimensional construct, which is meaningfully related to relevant variables. The results pave the way for further psychometric studies, as well as studies on how perceptions of external control of the home are associated with different aspects of health among people with PD. The HCQ instrument is foremost useful in research to further the understanding of housing and health dynamics in different sub-groups of the ageing population, for example, people with PD. Such knowledge has the potential to support the development of rehabilitation services, housing interventions and policies supporting active and healthy ageing in this specific population.

## Data Availability

The dataset for the current study is not publicly available due to ongoing longitudinal data collections. The data is stored at the Health Science Centre (Lund University, Sweden) and can be requested from Maria H. Nilsson (Principal Investigator; maria_h.nilsson@med.lu.se).
